# Falsification periods seen with an immediate reduction in Alzheimer's disease with semaglutide

**DOI:** 10.1002/alz.70150

**Published:** 2025-05-13

**Authors:** Eric Widera

**Affiliations:** ^1^ Department of Medicine, Division of Geriatric Medicine University of California San Francisco San Francisco California USA; ^2^ Geriatrics, Palliative and Extended Care San Francisco Veterans Affairs Healthcare System San Francisco California USA

1

Wang and colleagues recently published a target trial emulation study concluding that semaglutide is associated with significantly reduced risk for first‐time Alzheimer's disease diagnosis in patients with type 2 diabetes compared to patients prescribed other antidiabetic medications.^1^ Notably, this association, as seen in the Kaplan–Meier curves, emerged within 30 days of the first prescription of semaglutide, a finding that warrants several considerations.

First, the Kaplan–Meier curves, a graphical representation of time‐to‐event endpoints, appear to separate immediately after day 0, potentially even before individuals fill their prescriptions and take their first dose. Second, it is unclear as to by what biological mechanism semaglutide would reduce the progression to Alzheimer's disease within days as seen in the Kaplan–Meier curve, given that this is a disease that takes years to manifest and progress.

A more plausible explanation is the presence of residual confounding or confounding by indication, both of which call into question the study's results. As Mohyuddin and Prasad point out in a recent viewpoint, an early separation of the Kaplan–Meier curves is one mechanism for detecting residual confounding in observational studies.^2^ Early separation argues against a true treatment effect, and rather indicates that the two groups were not truly comparable from the start.

In addition, there is concern that the results of this trial act as a “falsification period” for other analyses conducted with the same dataset and methods. A “falsification period” is a time period during which no association between the intervention and the actual outcome is expected.^3^ For example, semaglutide is not expected to have an association with Alzheimer's disease within 30 days, nor for that matter are diseases like colon or liver cancer, which also take many months to years to develop. Yet, another publication from the authors using the same dataset and similar methods showed an immediate decrease in the incidence of colon cancer and liver cancers with semaglutide when compared to those prescribed insulin.^4^ The presence of falsification periods, as evidenced by both of these trials, should raise doubt about associations found between semaglutide and other outcomes using similar datasets and methods, including that seen with tobacco use disorder, cannabis use disorder, suicidal ideation, and alcohol use disorder.^5^, ^6^, ^7^, ^8^

Although we can be hopeful that interventions that address underlying risk factors for Alzheimer's disease will ultimately show benefits in preventing this devastating disease, this trial should be used to support this hypothesis given the above limitations.

## CONFLICTS OF INTEREST STATEMENT

E.W. has no disclosures.

## Supporting information




Supporting Information
